# Plasma photonic crystal ‘kaleidoscope’ with flexible control of topology and electromagnetism

**DOI:** 10.1007/s12200-024-00137-z

**Published:** 2024-10-17

**Authors:** Jing Wang, Shuang Liu, Weili Fan, Shuo Wang, Cuicui Lu, Yafeng He, Fucheng Liu, Xiaoyong Hu

**Affiliations:** 1 College of Physics Science and Technology, Hebei University, Baoding, 071002 China; 2 State Key Laboratory for Mesoscopic Physics and Department of Physics, Peking University, Beijing, 100871 China; 3 School of Physics, Beijing Institute of Technology, Beijing, 100081 China

**Keywords:** Plasma photonic crystals, Dielectric barrier discharges, Multi-freedom control, Topological state, Dynamic reconfiguration

## Abstract

Continuous development of photonic crystals (PCs) over the last 30 years has carved out many new scientific frontiers. However, creating tunable PCs that enable flexible control of geometric configurations remains a challenge. Here we present a scheme to produce a tunable plasma photonic crystal (PPC) ‘kaleidoscope’ with rich diversity of structural configurations in dielectric barrier discharge. Multi-freedom control of the PPCs, including the symmetry, dielectric constant, crystal orientation, lattice constant, topological state, and structures of scattering elements, has been realized. Four types of lattice reconfigurations are demonstrated, including transitions from periodic to periodic, disordered to ordered, non-topological to topological, and striped to honeycomb Moiré lattices. Furthermore, alterations in photonic band structures corresponding to the reconstruction of various PPCs have been investigated. Our system presents a promising platform for generating a PPC ‘kaleidoscope’, offering benefits such as reduced equipment requirements, low cost, rapid response, and enhanced flexibility. This development opens up new opportunities for both fundamental and applied research.

**Graphical Abstract:**

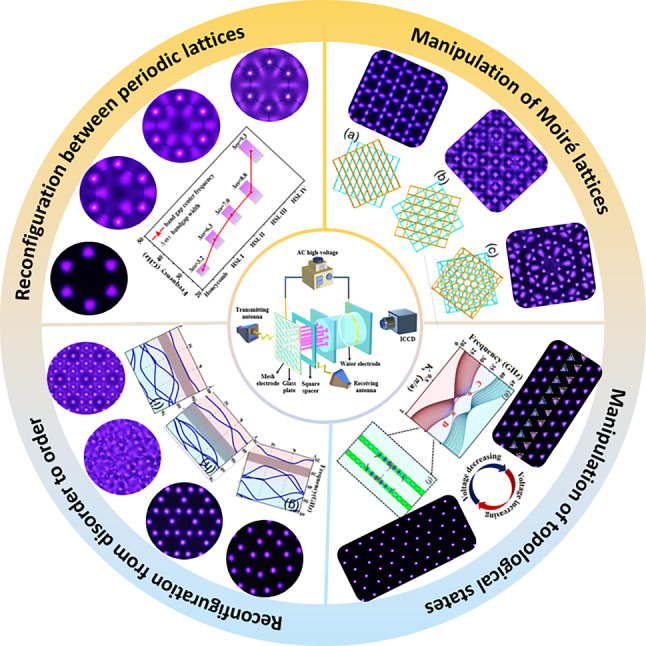

**Supplementary Information:**

The online version contains supplementary material available at 10.1007/s12200-024-00137-z.

## Introduction

**Fig. 1:**
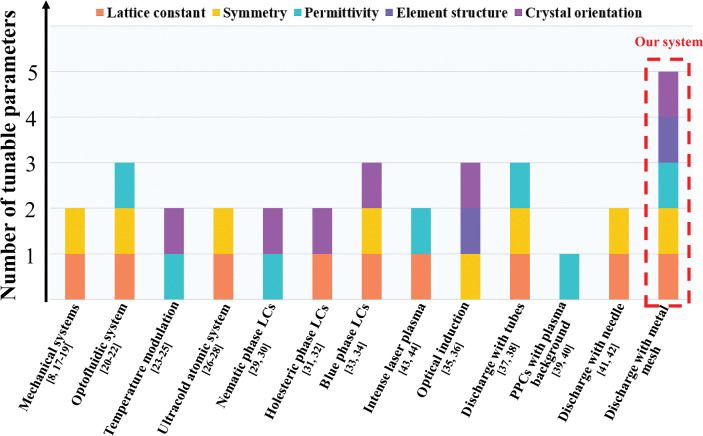
Control of tunable PCs in various systems

Photonic crystals (PCs) have captivated researchers in the field of photophysics due to their remarkable properties, ranging from negative refraction to light localization [1–6]. One intriguing aspect is the possibility of achieving tunable PCs, which would enable flexible control over their properties. This is essential because the specific requirements for PC geometry and material compositions vary across different applications [7–16]. Heretofore, a number of proposals for producing tunable PCs have been put forward, such as mechanical [8, 17–19], optofluidic [20–22], thermal [23–25], ultracold atomic [26–28], liquid crystal [29–34], optical induction [35, 36], and plasma systems [37–44]. The tunable responses of PCs are normally manipulated by five structural parameters: the lattice constant, crystal symmetry, dielectric constant, crystal orientation, and structure of scattering elements. Indeed, in a majority of photonic crystal systems, the ability to dynamically modulate structural parameters is limited to only a certain subset. This restriction results in a reduced degree of adjustability and flexibility. For instance, in mechanical systems as shown in Fig. 1, two parameters (2P) including the lattice constant and crystal symmetry can be tuned by compressing or stretching the materials [18, 19]. In optofluidic systems, the lattice constant, crystal symmetries, and dielectric constant (3P) can be modulated by manipulation of liquid filling position or substitution [20–22]. In thermal systems, the dielectric constant and crystal orientation (2P) can be tuned by utilizing temperature-sensitive materials [23–25]. In plasma systems composed of periodic arrays of discharge tubes, the lattice constant and dielectric constant (2P) are adjusted by changing the address electrode or modulating the plasma density [37, 38]. To date, while substantial progress has been made, achieving flexible control over photonic crystals, wherein all structural parameters can be dynamically tuned, remains a challenge. There is a requirement for the development of a robust fabrication strategy that enables the creation of highly flexible photonic crystals. Such advancements would open up a wide range of applications across various fields in science and technology.

In this paper, we propose and demonstrate an effective method to generate a tunable PPC ‘kaleidoscope’ with a rich diversity of geometric configurations in the dielectric barrier discharge system (DBD). Multi-freedom control of the PPCs including the symmetry, dielectric constant, lattice constant, structures of scattering elements, and crystal orientation (5P) have been realized. Four types of reconfigurations between lattices from periodic to periodic, from disordered to ordered, from non-topological to topological, and from striped to honeycomb Moiré lattices have been proposed. The reconstruction is fast with time response of only several seconds and is low-cost, which can be operated in ambient air. The variations in photonic band structures resulting from the reconfiguration of different PPCs have been investigated. Experimental measurements and numerical simulations are in good agreement. Our method opens a new avenue to investigate tunable plasma metamaterials in a controllable way, which can find wide applications in integrated optical components, signal processing, imaging, cloaking, wireless communications, etc.

## Experimental setup and theoretical framework

**Fig. 2:**
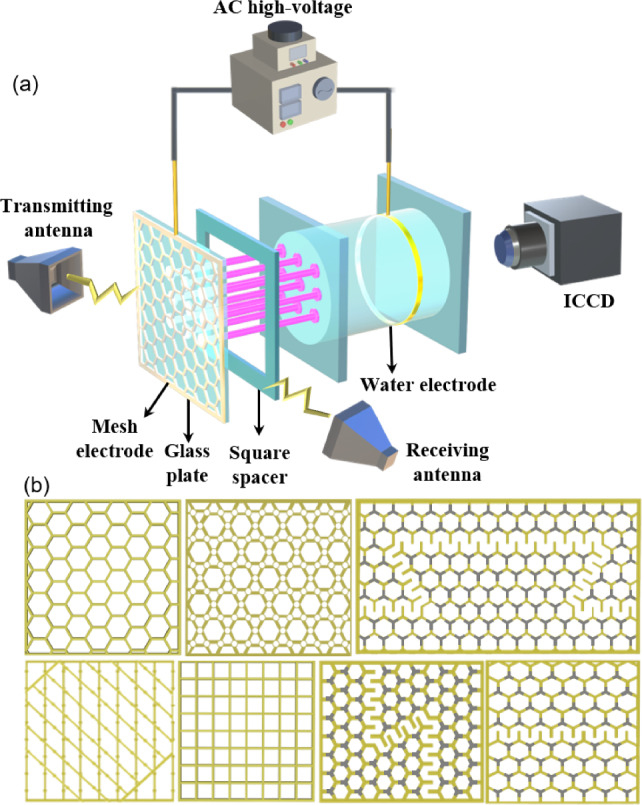
**a** Schematic diagram of the experimental device.** b** Schematics of mesh electrodes with different structures. In** b**, the yellow color indicates the metal electrode, while the grey color denotes the PTFE layer that covers the mesh electrode

As depicted in Fig. 2a, the water electrode on the right is made by filling tap water into a cylindrical container with an inner diameter of $80 \mathrm {~mm}$. It is sealed with glass plates varying in thickness within a range of 0.5–2 mm. The water not only acts as a conductor and coolant but also serves as a transparent medium for observing the discharges. A metal ring that is grounded is immersed in the water. The electrode on the left side is a metal mesh electrode, which is connected to an Alternating Current (AC) sinusoidal voltage. The amplitude of AC voltage can be adjusted within the range of 0 to $10 \mathrm {~kV}$, while the frequency can be modulated between 0 and $80 \mathrm {~kHz}$. The structure of mesh electrode can be varied in different ways. We present three types of mesh electrodes as illustrated in Fig. 2b, including (I) periodic arrays composed of different symmetries of unit cells such as square, hexagonal, and Kagome; (II) Moiré-like electrodes fabricated by rotating two identical mesh electrodes with different angles; (III) specially designed mesh electrodes by pasting polytetrafluoroethylene (PTFE) layers on certain nodes of the mesh. A square quartz frame with a thickness of 1–4 mm is sandwiched between the water and mesh electrode, serving as the lateral discharge boundary. The electrodes are placed in a large vacuum chamber which can be pumped and filled with different gas mixtures.

The electromagnetic transmission characteristics of PPCs are detected by microwave diagnostics. Microwave excitation is achieved using a millimeter-wave source and emitted via a broadband microwave horn antenna. On the other side, a pyramidal horn antenna receiver is placed to receive the transmitted signals. The distance between the transmitting and receiving horn antennas is approximately $35 \mathrm {~cm}$. The microwave with TM mode has been utilized. The band pass characteristics can be surmised from the measurements of transmittance spectra $\mathrm {S_{21}}$ (More details of experimental setup are provided in Fig. S1 in the Supplementary Materials).

The dispersion relations of PPCs are calculated by using COMSOL software under the Lorentz-Drude model. In accordance with the experimental measurement, we studied the photonic band diagrams under TM mode. The propagation of electromagnetic (EM) waves can be analyzed by Maxwell equations in the form of Helmholtz equation [45],

1 $$\begin{aligned} {\nabla \times \left[ \mu _{r}^{-1}({\varvec{r}}) \nabla \times {\varvec{E}}({\varvec{r}})\right] -k_{0}^{2}\left[ \varepsilon _{r}({\varvec{r}})-\text{j} \sigma /( \omega \varepsilon _{0})\right] {\varvec{E}}({\varvec{r}})=0}, \end{aligned}$$

where the dielectric conductivity $\sigma$ is 0, the permeability $\mu _{r}({\varvec{r}})$ is 1, and the wave vector $k_{0}=\omega / c$. The relative dielectric constant $\varepsilon _{r}({\varvec{r}})$ can be expressed as $\varepsilon _{\textrm{g}}$ in neutral gas $\left( \varepsilon _{\textrm{g}}=1\right.$) and $\varepsilon _{\textrm{p}}$ in plasma regions, written as [46]

2 $$\begin{aligned} \varepsilon _\textrm{p}=1-\omega _\textrm{p e}^{2} /\left( \omega ^{2}-\text{i} v_{m} \omega \right) , \end{aligned}$$

where $v_{m}$ denotes the electron elastic collision frequency, the plasma frequency $\omega _\textrm{p e}=\left( e^{2} n_{e} / (\varepsilon _{0} m)\right) ^{1 / 2}$. $n_{e}$ is the electron density which is generally in the order of $10^{13} \mathrm {~cm}^{-3}-10^{15} \mathrm {~cm}^{-3}$ depending on the discharge parameters. Based on Bloch’s theorem, each eigenvector ${\varvec{E}}({\varvec{r}})$ has the form as

3 $$\begin{aligned} \begin{aligned}&{\varvec{E}}({\varvec{r}})={\varvec{u}}_{\varvec{{k}}}({\varvec{r}}) \exp (\text{i} {\varvec{k}} \cdot {\varvec{r}}), \\&{\varvec{u}}_{\varvec{{k}}}({\varvec{r}})={\varvec{u}}_{\varvec{{k}}}\left( {\varvec{r}}+{\varvec{R}}_l\right) , \end{aligned} \end{aligned}$$

where ${\varvec{u}}_{\varvec{{k}}}({\varvec{r}})$ is a periodic function of the lattice and ${\varvec{R}}_l$ is the lattice vector. The eigenfrequencies have been calculated for each wave vector ${\varvec{k}}$ along the boundary of irreducible Brillouin zone and the photonic band diagrams can be obtained.

## Results and discussion

### Reconfiguration between periodic lattices

**Fig. 3:**
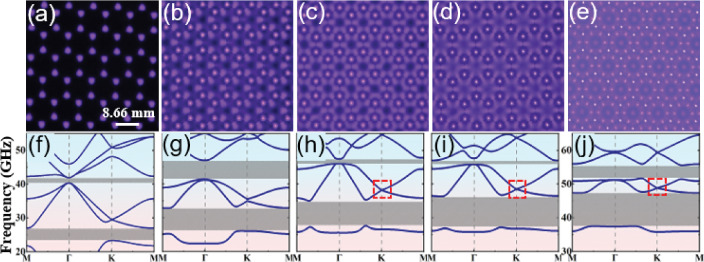
Reconfiguration from a simple honeycomb lattice to various honeycomb superlattices and the corresponding photonic band structures. ${\textbf{a}}$ *U* = 3.2 kV, simple honeycomb lattice. ${\textbf{b}}$ *U* = 4.0 kV, honeycomb superlattice I. ${\textbf{c}}$ *U* = 4.2 kV, honeycomb superlattice II. ${\textbf{d}}$ *U* = 4.4 kV, honeycomb superlattice III. ${\textbf{e}}$ *U* = 5.0 kV, honeycomb superlattice IV. Other experimental parameters are: the working gas is 100% ambient air, the gas pressure *p* = 114 Torr, the distance of gas gap *d* = 2.0 mm, and the frequency of supplied voltage *f* = 50 kHz. A honeycomb mesh electrode with lattice constant *a* = 8.66 mm is utilized. The electron elastic collision frequency $v_m$ = 40 GHz. The red rectangles indicate the positions of Dirac cones. The grey strips denote the positions of omnidirectional band gaps

We first examine the reconfiguration between lattices from periodic to periodic. Such PPCs are classical structures that possess photonic bandgaps (PBGs) and characterized by Bloch theory. Figure 3 shows the reconfiguration from a simple honeycomb lattice to various honeycomb superlattices with voltage increasing. At $U=3.2 \mathrm {~kV}$, a simple honeycomb lattice is formed. It is composed of discharge filaments ignited at the six vertexes of each honeycomb cell of the mesh electrode (Fig. 3a). At $U=4.0 \mathrm {~kV}$, new large triangular plasma elements are produced around each bright filament. We define this composite lattice as honeycomb superlattice I since it contains two different types of plasma elements that are the large triangular elements and small circular elements (Fig. 3b). As shown in Fig. 3c, d, further increasing the supplied voltage leads to more intriguing honeycomb superlattices. To our best knowledge, these superlattices are observed for the first time in gas discharge systems. The reconfiguration from the simple honeycomb lattice to honeycomb superlattices results in crystal symmetry reduction, giving rise to enlargement of band gap sizes. As illustrated in Fig. 3f–j, two OBGs can be obtained for these honeycomb-type lattices and the sizes of 1st OBG are enlarged significantly. The positions of 1st OBGs are located in the ranges of 23.5–26.7 GHz, 26.4–32.7  GHz, 27.8–34.8 GHz, 27.4–36.2 GHz, and 37.7–47.0 GHz, respectively, for the simple honeycomb lattice to honeycomb superlattice IV. The positions shift to the higher frequencies since a large number of filaments are produced when the supplied voltage is increased. Besides, Dirac cones at point *K* can be also obtained for honeycomb superlattices II, III and IV, which may bring out many unusual phenomena such as zero refraction, pseudospin-mediated vortices and conical diffraction [5, 47, 48]. The reconstruction between different lattices takes place very quickly within several seconds, which has good reversibility and reproducibility. A reversed reconfiguration can be obtained if the supplied voltage is decreased. Importantly, all these PPCs are created in simply $100 \%$ ambient air without particular treatment. The low-cost nature of this approach shows great potential for large-scale and customizable production in real-world applications. It is also worthy to point out that when the distance of the discharge gap has been changed, the lattice constant and the fundamental symmetry of plasma lattices will keep fixed, which primarily depend on the structure of mesh electrode, while the plasma elements can be varied with respect to their size, symmetry and fine structures. Clearly, our system serves as an excellent candidate for achieving reconfiguration between different periodic lattices, further expanding its applicability and versatility in various fields. The experimental diagnosis of band gaps as well as more examples of the reconfiguration between lattices from periodic to periodic can be found in Figs. S3–S5 in the Supplementary Material.

### Reconfiguration between lattices from disordered to ordered

**Fig. 4:**
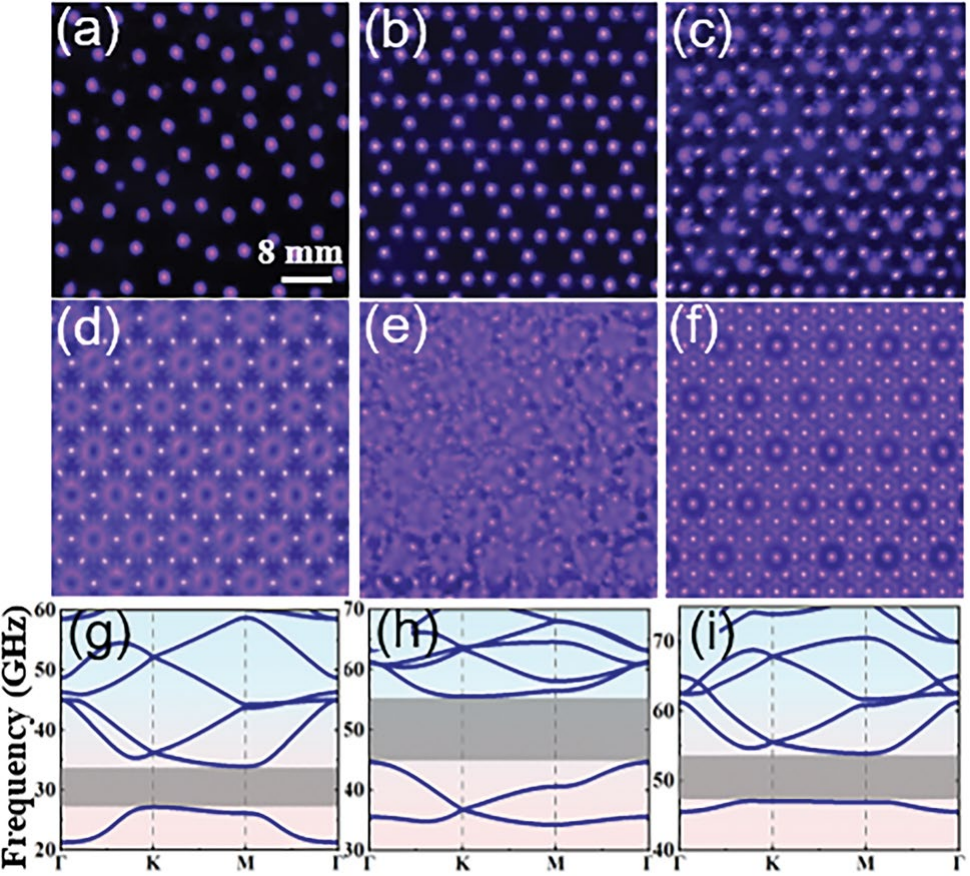
Reconstruction between lattices from disordered to ordered with increasing voltage. ${\textbf{a}}$ *U* = 3.5 kV, disordered lattice. ${\textbf{b}}$ *U* = 3.7 kV, ordered Kagome lattice. ${\textbf{c}}$ *U* = 3.9 kV, disordered honeycomb superlattice. ${\textbf{d}}$ *U* = 4.1 kV, ordered honeycomb superlattice I. ${\textbf{e}}$ *U* = 4.3 kV, disordered diffuse state. ${\textbf{f}}$ *U* = 5.3 kV, ordered honeycomb superlattice II. Other experimental parameters are: 100% ambient air, *p* = 114 Torr, *f* = 50 kHz, *d* = 2.0 mm. A Kagome mesh electrode with *a* = 8 mm is utilized. The band structures of **g–i** correspond to the lattices ${\textbf{b}}$, ${\textbf{d}}$, and ${\textbf{f}}$, respectively. $v_m$ = 40 GHz

We also realize controllable reconfiguration between lattices from the disordered to the well-ordered. As shown in Fig. 4, the plasma lattices transit from the disordered lattice—ordered Kagome lattice—disordered honeycomb superlattice—ordered honeycomb superlattice I—disordered diffuse state—ordered honeycomb superlattice II with increasing applied voltage. The system exhibits periodic transitions between disordered and ordered states. The band diagrams of the ordered Kagome lattice, honeycomb superlattice I and II have been calculated as shown in Fig. 4g–i. It is seen that the band gaps fall within the frequency ranges of 27.3–33.6 GHz, 44.8–55.1 GHz, and 47.4–53.6 GHz, respectively, which change significantly with structural reconfiguration of different PPCs. Note that the disordered lattices in our system can be classified into two categories: one is the steady disordered lattice, in which the distributions of filaments are disordered, but the whole lattice always keeps steady over time; the other is the dynamic disordered lattice, in which the distributions of filaments are disordered, meanwhile, the filaments exhibit dynamic behavior, which are moving or oscillating randomly in real-time. Here, the lattice shown in Fig. 4a is the steady disordered lattice, while the lattices in Fig. 4c and e are the dynamic disordered lattice. As is known, the field of disordered photonics has attracted increasing attention in recent years, ranging from the fundamental investigations such as topological insulator and Anderson localization [49, 50], to applications in random lasing [51], imaging [52], and solar energy [53]. It is proven that increasing the disorder of photonics can lead to many intriguing, and sometimes unexpected, physical phenomena. The dynamical control of the reconstruction from disordered to ordered plasma lattices herein may bring about new exploitable properties and capabilities for wide applications.

### Manipulation of Moiré lattices

**Fig. 5:**
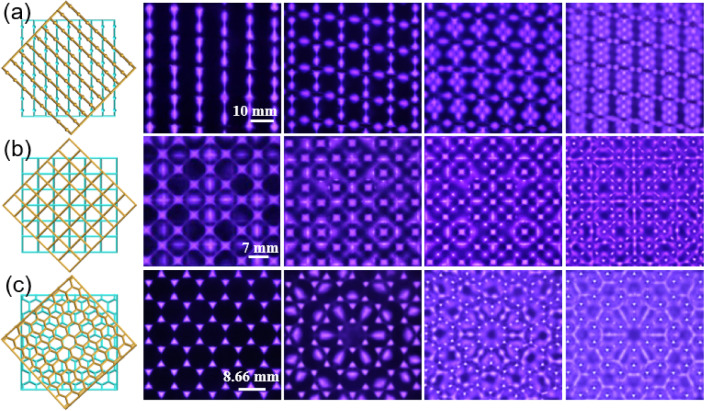
Manipulation of different Moiré lattices with increasing voltage. ${\textbf{a}}$ PPCs obtained by rotating two striped mesh electrodes. The ratio of Ar to Air in the mixture gas is 2:3, *p* = 190 Torr, *f* = 50 kHz, *d* = 2.0 mm. The lattice constant of the striped metal array *a* = 10 mm. ${\textbf{b}}$ PPCs obtained by rotating two square mesh electrodes. The ratio of Ar to Air in the mixture gas is 1:1, *p* = 152 Torr, *f* = 50 kHz, *d* = 2.0 mm. The lattice constant of the square metal array *a* = 8 mm. ${\textbf{c}}$ PPCs obtained by rotating two honeycomb mesh electrodes. The ratio of Ar to Air in the mixture gas is 1:1, *p* = 152 Torr, *f* = 50 kHz, *d* = 2.0 mm. The lattice constant of the honeycomb metal array *a* = 8.66 mm

Moiré lattices are composite structures formed by the superposition of two identical periodic structures [54–56]. Due to the interplay between two sub-lattices, Moiré lattices exhibit extraordinary properties that single-layer materials do not possess. For instance, larger band gaps [57], easier light location [56], topological states [58], and tunable photon speed [56] can be realized in Moiré lattices by simply modulating the Moiré angle. At present, Moiré lattices in photonics are normally fabricated by writing two periodic lattices into the same crystal, or by twisting bilayer photonic crystal slabs. Here we propose a new method for yielding controllable Moiré PPCs by rotating two identical mesh electrodes in DBD. As shown in Fig. 5, a rich variety of Moiré lattices have been obtained by using two twisted striped, square, and honeycomb lattices, respectively. As the supplied voltage increases, Moiré PPCs will reconstruct from the simple lattices to various complex superlattices, leading to great changes in optical properties. In particular, the rotation angle between these two twisted electrodes can be modulated freely. When the mesh electrode has been rotated, the positions of filaments will be changed correspondingly, resulting in a variety of new plasma lattices. This provides a new degree of freedom to manipulate the structures of Moiré PPCs. Since these plasma lattices are luminous and can be easily tracked, our system provides an effective way for studying the fundamental properties of Moiré lattices, which may offer insights for other systems such as condensed matter and cold atoms.

### Manipulation of topological states

**Fig. 6:**
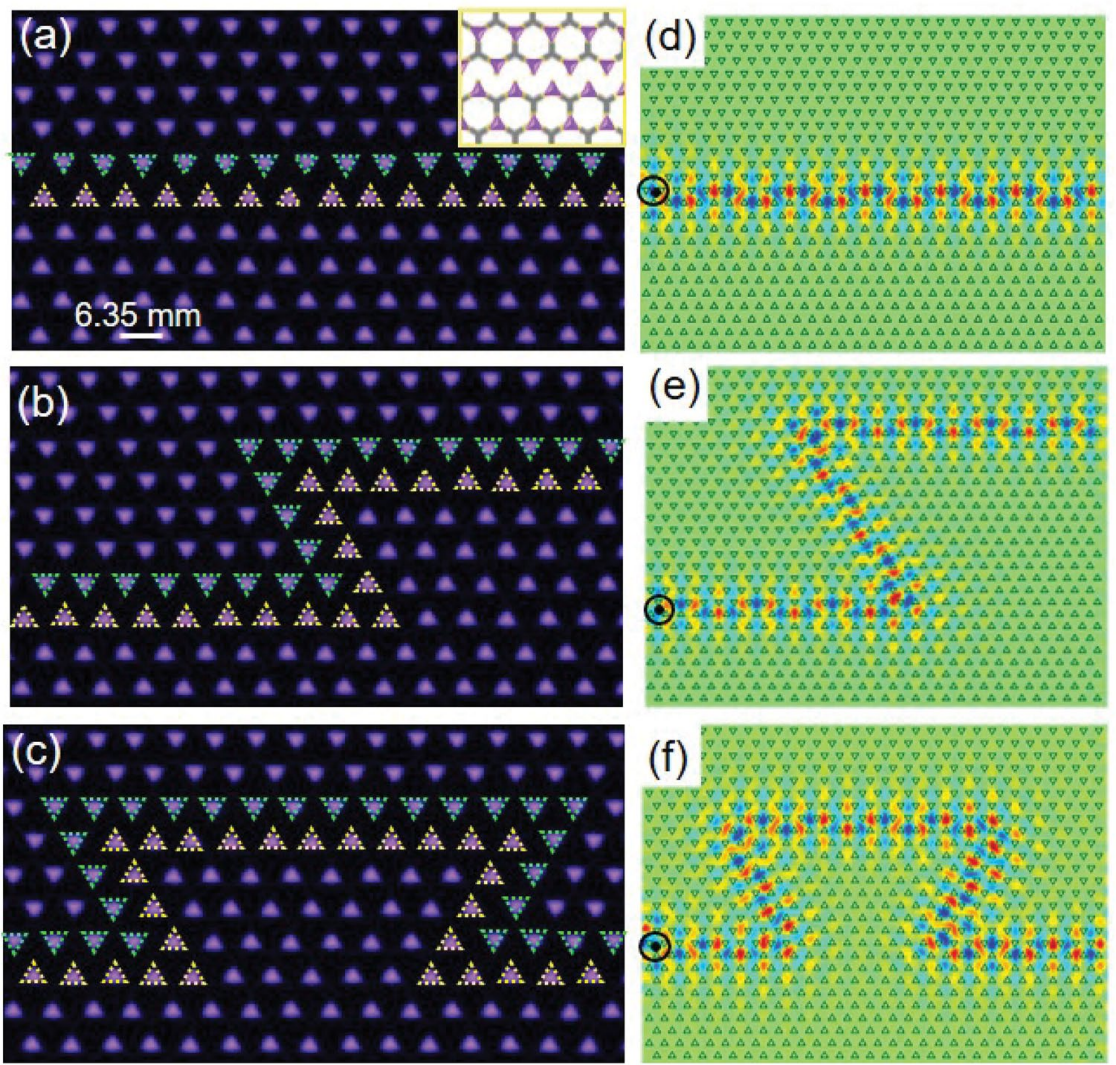
Robustness of topological edge states. **a**–**c** Snapshots of Line-type, Z-type and $\Omega$-type interface topological PPCs. *U* = 3.6 kV, 3.6 kV, 3.6 kV, respectively. The ratio of Ar to Air in the mixture gas is 3:2, *p* = 135 Torr, *f* = 50 kHz, *d* = 2.0 mm, the exposure time $\Delta t$ = 1/10 s. The green and yellow triangles denote the triangular plasma elements of opposite orientations at the interface, the triangle side length is 3.0 mm. **d**–**f** Unidirectional propagation of the edge states localized at the interface. The inset in ${\textbf{a}}$ shows the positions of plasma elements on the mesh electrode

Photonic topological insulators are a new phase of matter with the striking property that photon transmission occurs only on their surfaces. They were first proposed by Haldane and Raghu, and demonstrated experimentally by Wang et al., using a microwave scale gyromagnetic PC which is a photonic analog of a Chern insulator [59–61]. So far, there have been some proposals to realize topological photonics, such as waveguide arrays and coupled ring resonators [62, 63]. Recently, Yuan et al. constructed different valley PPCs by arranging plasma tubes into a gas background, or in reverse, by embedding an array of dielectric rods into a plasma background. By modulating the electron density, they obtained tunable topological states [39, 64]. Nevertheless, it is still difficult to yield a reconfigurable topological PPC whose geometric configurations can be tuned dynamically. Here we present an effective method for generating topological PPCs with strong robustness and tunability. As shown in Fig. 6, the Line-type, Z-type and $\Omega$-type interfaces in triangular lattice PPCs with $C_{3}$ symmetry have been obtained. It is realized by using a specially designed honeycomb mesh electrode, which is fabricated by placing PTFE coatings at specific sites (Fig. 2b and Figs. S1, S2). As the supplied voltage is increased, the electrical potentials at the vertexes of the honeycomb mesh electrode, where there are no PTFE coatings, are the largest, leading to formation of triangular plasma elements at these positions. Inspection of voltage-current waveforms (Fig. $\textrm{S} 6$ in Supplementary Materials) reveals that only one single current pulse appears per half period of the supplied voltage. This indicates that all the plasma elements of topological PPCs are ignited synchronously, occurring within a time window of $350 \mathrm {~ns}$. They have rather a good temporal periodicity, which repeats in the same manner in each subsequent half-period with the jitter less than $30 \mathrm {~ns}$. Such features enable active spatial and temporal control over topological PPCs. First, the orientation of triangular plasma elements can be manipulated by the distribution of PTFE layer. Second, the plasma density (permittivity) and the sizes or shapes of plasma elements can be adjusted by altering the supplied voltage and gas pressure. Third, the good temporal periodicity allows one to control the formation of topological PPCs temporally by modulating the voltage frequency.

In order to demonstrate the unidirectional propagation of the edge states localized at the interface, we set a point source on the left side of intersection as shown in Fig. 6d–f. We define the topological PPCs with two different orientations of triangular elements as type-A with $\alpha =-30^{\circ }$ and type-B with $\alpha =30^{\circ }$, respectively. One can see that the electromagnetic energy is perfectly transmitted forward along the interface for all the Line-type, Z-type and $\Omega$-type topological PPCs, despite the presence of sharp corners. The backscattering of electromagnetic waves has been suppressed [65]. This suggests the good unidirectional propagation properties of our topological PPCs, which can be used to manipulate microwave radiation for wide applications [66–69] (Fig. S9 in the Supplementary Material).

**Fig. 7:**
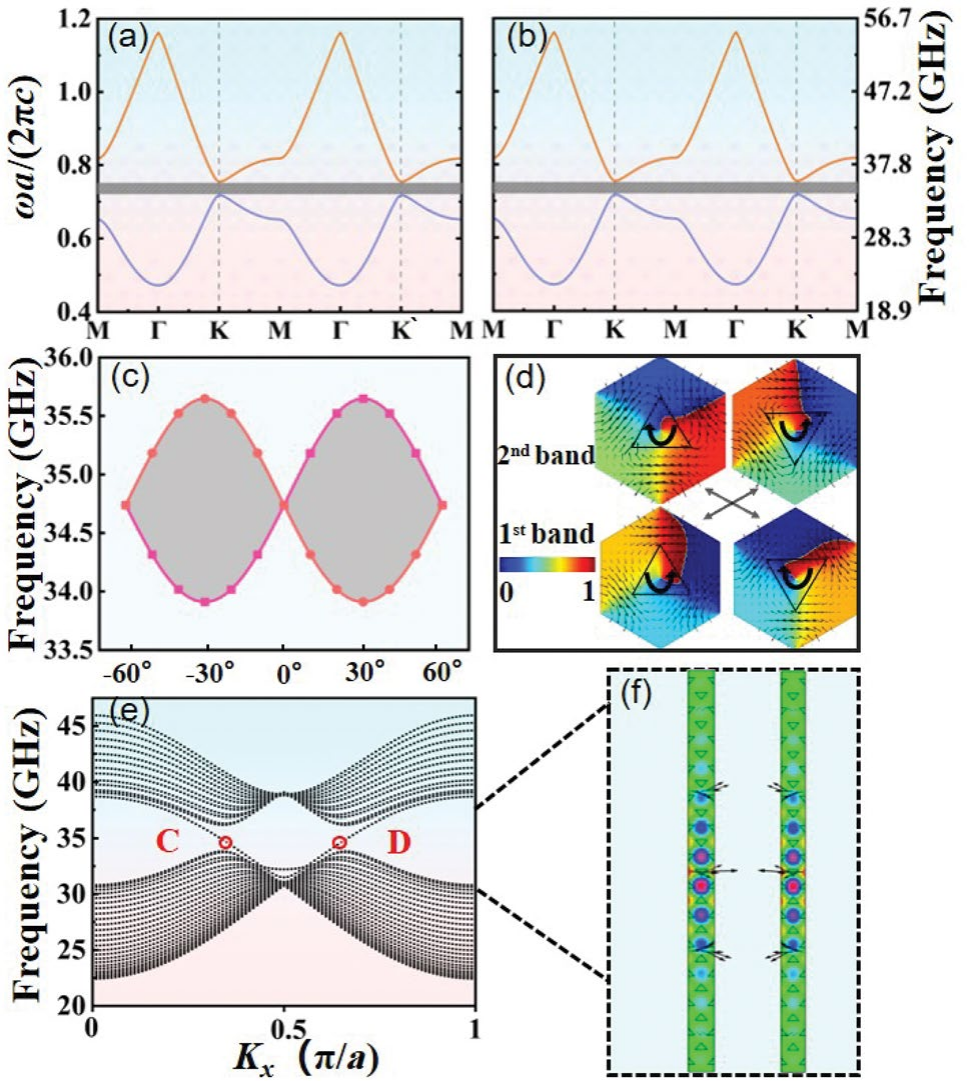
Photonic band structures and the vortex properties of eigenmodes. ${\textbf{a}}$ and ${\textbf{b}}$ Band diagrams of type-A and type-B triangular PPCs. ${\textbf{c}}$ The frequency variation of *K* valley during a cycle. ${\textbf{d}}$ Phase distribution and Poynting vector at *K* valley for type-A PPCs and type-B PPCs. ${\textbf{e}}$ The project band structure of supercell. ${\textbf{f}}$ $E_z$ distribution at the wave vector $k_x = 0.35\uppi /a$ and $k_x = 0.65\uppi /a$, respectively. The electron density of plasma $n_e=2.0\times 10^{14} \mathrm {~cm}^{-3}$, $v_m$ = 34 GHz

Figure 7 presents a detailed characterization of the topological properties of our PPCs. It is seen that a topological gap that locates in the range of 33.9–35.6 GHz forms in $\textrm{K}-\textrm{M}$ direction for both type-A and type-B PPCs as shown in Fig. 7a, b. The variation of valley frequency with different rotation angles of triangular plasma elements is shown in Fig. 7c. When $\alpha =m \uppi / 3$, with *m* being an integer, both $C_{3 v}$ symmetry and time-reversal symmetry are preserved, resulting in the formation of Dirac cones at the high symmetric points *K* and $K^{\prime }$. This is due to the perfect match of the mirrors of individual plasma elements to those of the triangular-lattice. Except for these specific angles, the mirrors are mismatched and the symmetry reduces to $C_{3}$ [70, 71]. In this case, the Dirac cones at *K* and $K^{\prime }$ points are opened, and a topology band gap can be generated. This produces a pair of frequency extrema in momentum space, known as valley states. At the $K\left( K^{\prime }\right)$ points, the band structures have phases characterized by either left- or right-handed polarization. The orbital angular momentum at the valley exhibits circular polarization, described by topological charge [64]. In this study, we concentrate on the properties of valley states at *K* point, while those for inequivalent $K^{\prime }$ point can be obtained through the time-reversal (TR) symmetry.

The topological charge has the form

4 $$\begin{aligned} l=\frac{1}{2 \uppi } \oint _{L} \nabla \left[ \arg \left( E_{z}\right) \right] \text{d} \vec {s}, \end{aligned}$$

where $\arg \left( E_{z}\right)$ denotes the phase of electric field, *L* represents the closed path around the singularity of electric field distributions. Topological charge characterizes the vortex properties of eigenmodes, indicating the number of times the phase changes from 0 to $2 \uppi$. If the phase undergoes a positive change of one cycle along the closed path (anticlockwise), it corresponds to a topological charge of $+1$. Conversely, if it is negatively changes by one cycle (clockwise), it corresponds to a topological charge of $-1$. As shown in Fig. 7d, at $\alpha =-30^{\circ }$, one can observe the left-handed circular polarized angular momentum (LCP, $l=-1$) for the higher frequency at *K* valley as well as the right-handed circular polarized angular momentum (RCP, $l=+1$) for the lower frequency at the same valley. At $\alpha =+30^{\circ }$, the right-handed circular polarized angular momentum (RCP, $l=+1$ ) for the higher frequency at *K* valley and the left-handed circular polarized (LCP, $l=-1$ ) angular momentum for the lower frequency at *K* valley can be obtained.

To elucidate the characteristics of Dirac degeneracy, we studied the field patterns for the two degenerate states at *K* point [72]. For the electromagnetic wave of TM mode, $E_{z}$ exists in *x*–*y* plane. The time-averaged Poynting vector is defined as [73]

5 $$\begin{aligned} {\varvec{P}}=\frac{1}{2} \times \left( {\varvec{E}} \times {\varvec{H}}^{*}\right) , \end{aligned}$$

where $P_{x}=-0.5 \times E_{z} \times H_{y}$, $P_{y}=0.5 \times E_{z} \times H_{x}$. The small surrounding arrows in Fig. 7d indicate the directions and magnitudes of the Poynting vector. The black arrow inside of the triangular element gives the direction of Poynting vector. One can see that the direction of Poynting vector is clockwise when the topological charge is +1. It is anticlockwise when the topological charge is $-1$. Obviously, the direction of Poynting vector is in good accordance with the value of topological charge.

As aforementioned, splicing the PPC structures of type-A and type-B together supports a valley-dependent topological edge state. Figure 7e, f show a schematic of the supercell together with its project band structure. The Floquet conditions are utilized to the boundary in *x*-direction and the scattering boundary conditions are adopted in *y*-direction. When the wave vector is fixed to $k_{x}=0.35 \uppi / a$ (point $\textrm{C}$) and $0.65 \uppi / a$ (Point $\textrm{D}$), the electric field mostly localizes at the interface of two structures and decays rapidly in the interior. Moreover, the directions of Poynting vectors of the edge states at C, D points are opposite. These suggest the formation of topological edge states in our PPCs.

**Fig. 8:**
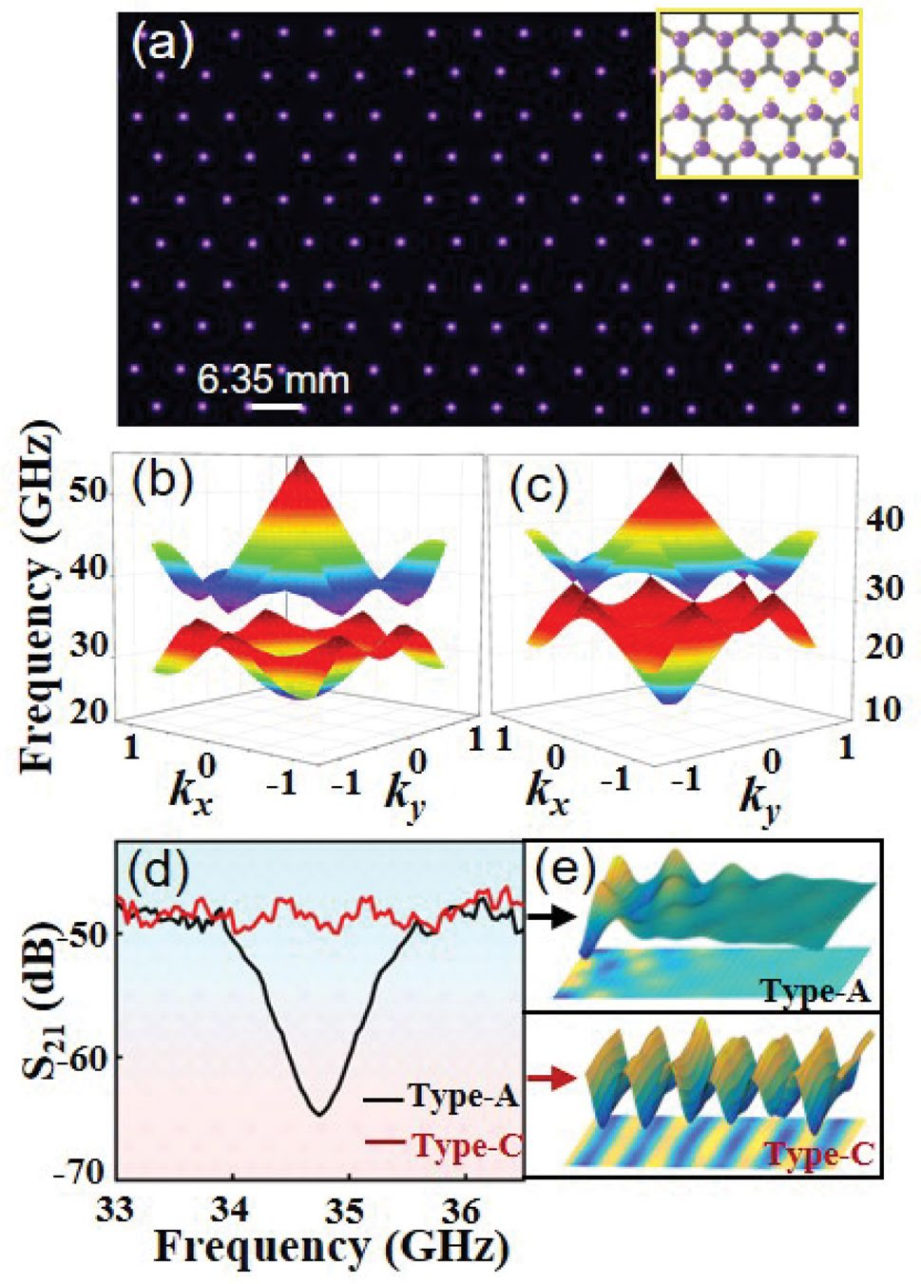
Triangular PPC and its band structure. ${\textbf{a}}$ Snapshot of the triangular PPC composed of circular plasma elements (defined as type-C PPC). *U* = 3.8 kV. The ratio of Ar to Air in the mixture gas is 3:2, *p* = 135 Torr, *f* = 50 kHz, *d* = 2.0 mm, the exposure time $\Delta t$ = 1/10 s. The diameter of the circular filaments is 1.2 mm. ${\textbf{b}}$ and ${\textbf{c}}$ 3D photonic bands of type-A (or B) PPC and type-C PPC, respectively. The average electron density $n_e=2.7\times 10^{14} \mathrm {~cm}^{-3}$, $v_m = 34$ GHz. ${\textbf{d}}$ Experimental transmittance spectra of millimeter waves for type-A (or B) PPC and type-C PPC. ${\textbf{e}}$ Simulated $E_z$ distribution for type-A (or B) PPC and type-C PPC with the frequency of incident microwave *f* = 34.7 GHz. A normalized colorbar is adopted

One of the most critical properties of our PPCs is the controllable reconfiguration from a topological PPC composed of different elements to a conventional PPC composed of identical elements. As depicted in Fig. 8, when increasing the applied voltage from $U=3.6 \mathrm {~kV}$ (Fig. 6a) to $3.8 \mathrm {~kV}$ (Fig. 8a), the topological PPCs composed of triangular elements with two opposite orientations will change to a simple triangular lattice composed of identical circular elements. We define this triangular PPC as type-C. Note that only the voltage is changed, while the other discharge parameters involving the structure of mesh electrode keep fixed. With this reconfiguration, the band gaps change from opening (Fig. 8b) to closing (Fig. 8c), and Dirac cones are formed at *K* valley and $K^{\prime }$ valley at the frequency $f=31.9 \mathrm {~GHz}$. The lattice symmetry is raised from $C_{3}$ to $C_{6 v}$, while the lattice constant remains invariant. The experiential verification of the photonic band gaps for type-A (or B) PPC and type-C PPC has been provided in Fig. 8d. One can see that a band gap can be observed in range of $34.3-35.4 \mathrm {~GHz}$ for type-A PPC, while no band gap forms in this range for type-C PPC. Experimental measurements are in good agreement with the theoretical calculations. As shown in Fig. 8e, if the frequency of incident microwaves locates in the band gap, for instance $f=34.7 \mathrm {~GHz}$ in type-A PPC, the microwaves will attenuate strongly. By contrast, the microwaves can propagate through the plasma lattice successfully with low-loss for type-C PPC.

## Conclusion

We present experimentally the realization of a flexibly controlled PPC ‘kaleidoscope’ with tunable topology and electromagnetism in DBD. Four types of reconfigurations between the lattices from periodic to periodic, from disordered to ordered, from non-topological to topological, and from striped to honeycomb Moiré lattices have been demonstrated. Multi-freedom control of PPCs including the symmetry, dielectric constant, crystal orientation, lattice constant, topological state, and structures of scattering elements has been achieved. The reconstruction process is rapid, with a time response of just a few seconds, and cost-effective, which is beneficial for largescale production. We believe that the unique design of mesh-water electrodes is the crucial factor responsible for yielding such rich diversity of plasma lattices. The mesh electrodes introduce two-dimensional periodic potential to provide a constrained lattice constant and symmetry for the plasma lattices, while due to the high nonlinear effects of our DBD system, these filaments are able to self-organize into various structures, even complex superlattices. On the other hand, the water not only serves as the conductor but also the coolant medium to ensure the high stability of the lattices. Both numerical and experimental investigations have been carried out to study the changes in photonic band structures resulting from lattice reconstruction. Furthermore, the valley-dependent topological edge states of our photonic crystal systems have been characterized. Our approach offers a promising platform for developing tunable plasma metamaterials with substantial flexibility, potentially opening up numerous possibilities for applications in integrated optical components, energy harvesting, imaging, signal processing, and cloaking.

## Supplementary Information

Below is the link to the electronic supplementary material.

Supplementary file1 (PDF 873 KB)

## Data Availability

The data that support the findings of this study are available from the corresponding authors, upon reasonable request.
