# Supplementary material for: Plasma photonic crystal ‘kaleidoscope’ with flexible control of topology and electromagnetism
Source: Front Optoelectron. 2024 Oct 17;17(1):34. doi: 10.1007/s12200-024-00137-z (PMC11485005; doi:10.1007/s12200-024-00137-z)
Supplement: Supplementary file 1 — Supplementary file1 (PDF 873 KB) [file 12200_2024_137_MOESM1_ESM.pdf]

**Supplementary materials：Plasma photonic crystal 'kaleidoscope' with flexible control of topology and electromagnetism**

Jing Wang[1], Shuang Liu[1], Weili Fan[1*], Shuo Wang[1], Cuicui Lu[3], Yafeng He[1], Fucheng Liu[1*], and Xiaoyong Hu[2*]

1 College of Physics Science and Technology, Hebei University, Baoding 071002, China

2 State Key Laboratory for Mesoscopic Physics and Department of Physics, Peking University, Beijing 100871, China.

3 School of Physics, Beijing Institute of Technology, Beijing, 100081, China

Corresponding author: fanweili@hbu.edu.cn, hdlfc@hbu.edu.cn, and xiaoyonghu@pku.edu.cn.

## 1. Experimental setup

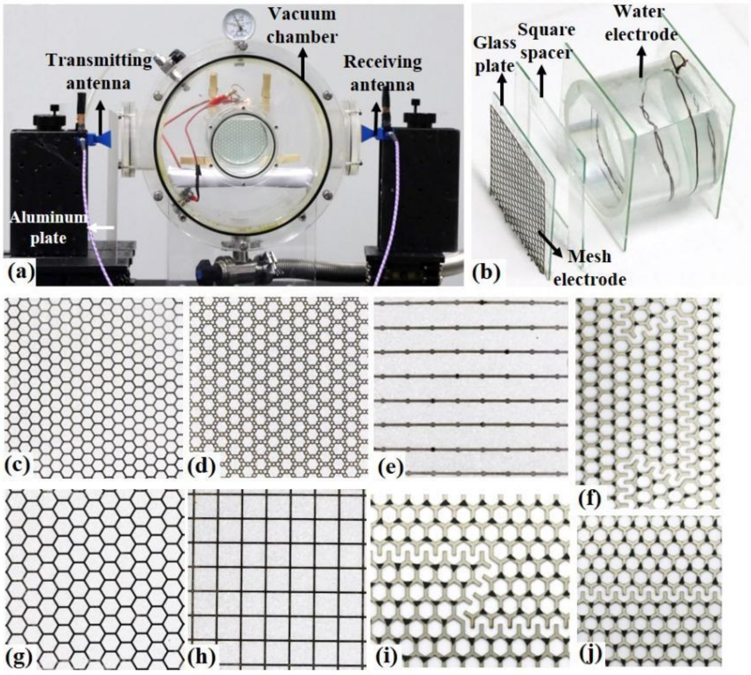


**FIG. S1.** Images of real experimental devices. (a) Front view of whole discharge cell. (b) Images of mesh-water electrodes. (c)-(j) Different types of metal mesh electrodes. The black triangles in (f), (i), (j) denote the polytetrafluoroethylene (PTFE) layers pasted on certain nodes of the mesh electrode so as to prevent discharges at these positions.

Figure S1 gives the images of real experimental devices. The discharge cell is placed in a large vacuum chamber that can be pumped and filled with a gas mixture. The water electrode is made by filling tap water into a cylindrical container sealed with quartz glass layers. The water also serves as a coolant and transparent medium for the observation and measurement of discharge filaments. The mesh electrode is fabricated by covering a quartz glass layer on a metal mesh composed of different cells. It is connected to a sinusoidal Alternating-Current (AC) power supply with the frequency range of 0-80 kHz and amplitude range of 1-10 kV. The mesh electrode introduces a two-dimensional Laplacian electric field to provide a constrained symmetry and lattice constant of plasma lattices, which is key for yielding controllable and stable plasma lattices. If the geometry of mesh electrode has been changed as shown in Figs. S1(c)-

(j), the symmetry of plasma lattices will be varied correspondingly. A square glass spacer is placed between two parallel electrodes, serving as a lateral boundary. When the applied voltage is increased to the discharge threshold, the filaments will be ignited in the gas gap and construct into different lattices with the changes of discharge parameters. An intensified charge-coupled device (ICCD, Andor DH334T) is employed to measure the spatiotemporal dynamics of the discharges at the end of water electrode. A digital phosphor oscilloscope (Tektronix TDS3054B) is utilized to record the voltage and current waveforms of the discharges, which are detected using a high-voltage probe (Tektronix P6015A 1000×) and a current probe (Tektronix TCP0030A).

The electromagnetic transmission characteristics of PPCs are detected by using microwave diagnostics. The microwaves were excited at a millimeter wave source (Agilent Vector Network Analyzer, ZNB40, 10.0 MHz–40.0 GHz) and launched from a broadband microwave horn antenna (18.0–26.5 GHz, XB-GH42-20K, and 26.5–40 GHz, XB-GH28-20K). An aluminum aperture plate with an opening of 10 cm × 2 mm is placed adjacent to discharge cell to reduce microwave absorption of the water electrode. A pyramidal horn antenna receiver (18.0–26.5 GHz, XB-GH42-20K, and 26.5–40 GHz, XB-GH28-20K) is placed on the other side to receive the transmitted signals. The microwave with the transverse magnetic (TM) mode has been utilized. The distance between the transmitting and receiving horn antennas is about 35 cm. Band pass characteristics can be surmised from the measurements of transmittance spectra of $S_{21}$ parameter.

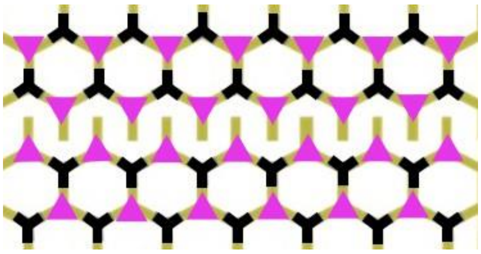

**FIG. S2.** Schematics of the discharges distributed on the mesh electrode. The black color indicates the PTFE coatings. The purple triangles denote the triangular plasma elements that have two different rotation directions.

In order to control the directions of triangular plasma elements as shown in Fig. 6 in the manuscript, we have specially designed the honeycomb mesh electrodes, which are fabricated by placing PTFE coatings at specific sites. As shown in Fig. S2, the cross points marked by the black color show the positions covered by PTFE layers, where the discharges are inhibited. As the supplied voltage is increased, the electrical potentials at the cross points of the honeycomb mesh electrode, where there are no PTFE coatings, are the largest, leading to the formation of triangular plasma elements at these positions (denoted by the purple triangles). Therefore, by setting the PTFE layer at the appropriate positions of the cross points, two kinds of triangles with different rotation angles can be obtained. Moreover, one can also design the shapes of mesh electrodes as Line-type, Z-type and Ω-type to form different transmission boundaries in the manuscript (Fig. 2(b)).

## 2. Reconstruction between different periodic lattices

Reconfiguration between different periodic lattices can be realized by readily changing the discharge parameters. Fig. S3 illustrates the reconstruction from a square lattice to various Lieb superlattices with voltage increasing. A square mesh electrode shown in Fig. 1S(h) is utilized here. At $U = 3.5$ kV, the filaments located at four vertexes of square cells are ignited and a simple square lattice is produced (Fig. S3(a)). At $U = 3.7$ kV, a Lieb lattice appears with addition of a new filament at the edge-center site of each square unit cell (Fig. S3(b)). A unique Dirac-flat band structure can be obtained in a Lieb lattice that may host various exotic properties, such as Klein tunneling[1], superconductivity[2], and ferromagnetism properties[3], [4]. At $U = 4.1$ kV, a novel Lieb superlattice I is achieved with presence of star-like elements on base of Lieb-5 lattice (Fig. S3(c)). When the voltage is increased to 4.9 kV, Lieb superlattice II forms, in which a new smaller square emerges inside of each unit cell of Lieb-5 lattice (Fig. S3(d)). Further increasing the applied voltage leads to a complex Lieb superlattice III. Here, an additional filament is ignited in the center of each unit cell, based on the framework of Lieb superlattice II (Fig. S3(d)). Obviously, all above plasma lattices are

well-defined periodic structures with high spatial symmetry. Our system allows for the dynamic reconstruction between different periodic lattices.

As respected, the band structures change significantly with reconfiguration of different ‘kaleidoscope’ PPCs. As shown in Figs. S3(f)-(j), no omnidirectional band gaps (OBGs) can be obtained for the simple square lattice and Lieb lattice, while the OBGs located in the ranges of 29.0-32.9 GHz, 34.5-43.5 GHz and 25.9-37.4 GHz are achieved for Lieb superlattice I to III, respectively. Besides, the Lieb superlattices possess larger OBGs due to the reduction of crystal symmetry. The controllable reconfiguration enables real-time regulation of photonic band structures, which can find wide applications in integrated optical components, photonic microwave filter, wireless, communications, and signal processing.

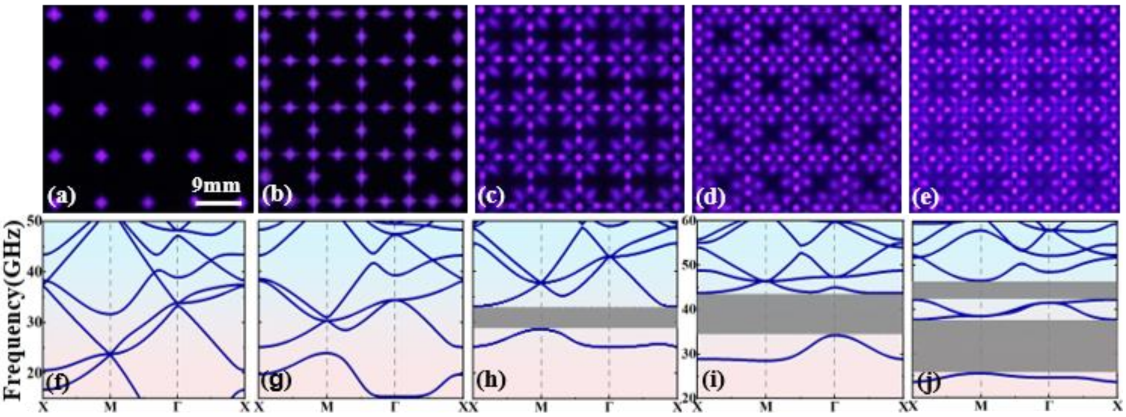


**FIG. S3.** Reconfiguration from square lattice to various Lieb superlattices with voltage increasing. (a) Square lattice, $U = 3.5$ kV; (b) Lieb lattice, $U = 3.7$ kV; (c) Lieb superlattice I, $U = 4.1$ kV; (d) Lieb superlattice II, $U = 4.9$ kV; and (e) Lieb superlattice III, $U = 5.3$ kV. The working gas is 100% ambient air. The gas pressure $p = 114$ Torr, and the gas gap $d = 2.0$ mm. The frequency of the supply voltage $f = 50$ kHz. A square mesh electrode with lattice constant $a = 9$ mm is utilized as shown in Fig. S1(h). The bottom panel gives the corresponding photonic band diagrams. The electron elastic collision frequency $v_m = 40$ GHz in simulation communications.

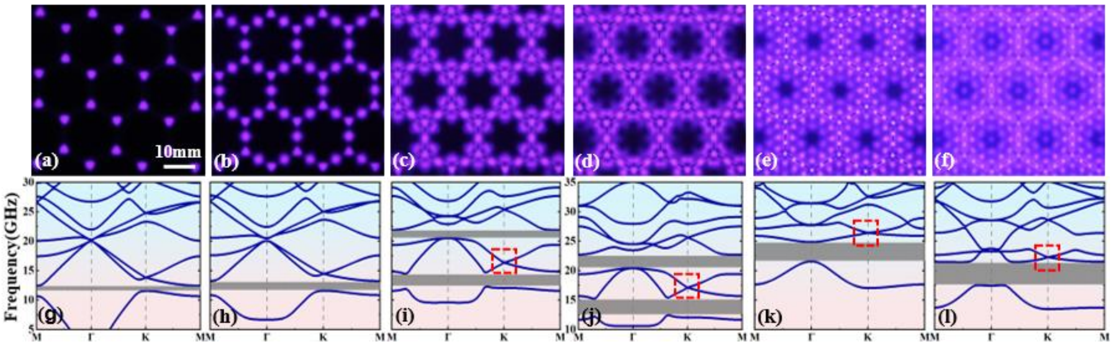


**FIG. S4.** Reconfiguration from a simple honeycomb lattice to different honeycomb superlattices with voltage increasing. (a) Simple honeycomb lattice, $U$ = 3.8 kV. (b) Honeycomb superlattice I, $U$ = 4.0 kV. (c) Kagome superlattice I, $U$ = 4.2 kV. (d) Kagome superlattice II, $U$ = 4.8 kV. (e) Honeycomb superlattice II, $U$ = 5.6 kV. (f) Honeycomb superlattice III, $U$ = 6.0 kV. Working gas: 100% ambient air. $p$ = 91 Torr, $d$ = 2.0 mm, $f$ = 50 kHz. A honeycomb mesh electrode with $a$ = 17.3 mm is utilized as illustrated in Fig. S1(g). The bottom panel shows the corresponding photonic band diagrams. $v_m$ = 36 GHz. The dashed red rectangles indicate the positions of Dirac cones.

We next demonstrate another example of the reconfiguration between different periodic lattices. If a honeycomb mesh electrode has been utilized, a simple honeycomb lattice can be reconstructed into complex honeycomb superlattices with voltage increasing. As shown in Fig. S4(a), when the discharge is just ignited at $U$ = 3.8 kV, a simple honeycomb lattice is produced, which is composed of triangular plasma elements located at the six vertexes of hexagonal cells. At $U$ = 4.0 kV, a honeycomb superlattice I is formed with addition of a new filament at the center of each edge (Fig. S4(b)). It has been proven that such a honeycomb superlattice can represent a hybrid fermionic and bosonic system, which contains pseudospin -1/2 and pseudospin -1 Dirac cones as well as a flat band in its band structure[5]. Further increasing the applied voltage leads to two types of intriguing Kagome lattices, which appear as a mesh structure braided by plasma lines (Figs. S4(c)-(d)). As the applied voltage is increased furthermore, two additional honeycomb superlattices can be obtained (Figs. S4(e)-(f)), which are much more complicated with emergence of a large number of filaments.

Similarly, the reconfiguration of honeycomb lattices leads to significant changes

in band structures. One can see from Figs. S4(g)-(l) that the size of OBGs is increased with the reconstruction from the simple honeycomb lattice to honeycomb superlattice III. Two OBGs can be achieved in Kagome superlattices, while only one OBG appears in honeycomb-type lattices. Moreover, Dirac cones can be obtained in Figs. S4(i)-(l), which may offer new capabilities and exploitable properties.

## 3. Microwave diagnostics of photonic band gaps

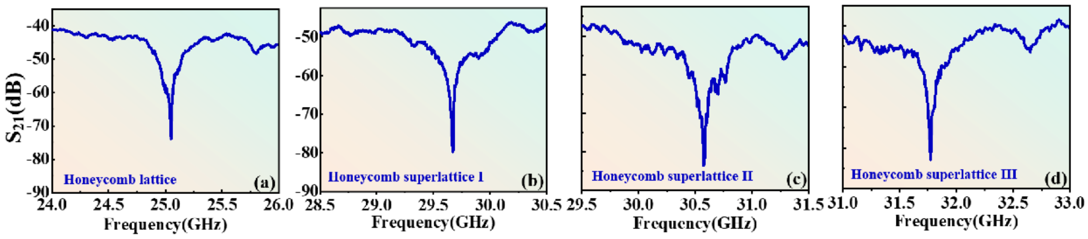


**FIG. S5.** Experimental transmittance spectra $S_{21}$ corresponding to the 1$^{st}$ OBGs presented in Figs. 3(a)-(d) in the manuscript.

Figure S5 shows the experimental diagnostics of the 1$^{st}$ OBGs corresponding to the honeycomb lattices presented in Figs. 3(a)-(d) in the manuscript. It is seen that the bandgaps locate at 25.1 GHz for the simple honeycomb lattice, while locating at 29.7 GHz, 30.6 GHz, and 31.8 GHz, respectively, for honeycomb superlattices I to III. The experimental measurements are well consistent with the calculation results in Figs. 3(f)-(i). Owing to the limitation of our microwave apparatus that the stop frequency is 40 GHz, the diagnosis of 1$^{st}$ OBG for honeycomb superlattice IV, whose central frequency is higher than 40 GHz, will be performed in the future.

## 4. Time-resolved measurements of topological triangular PPCs

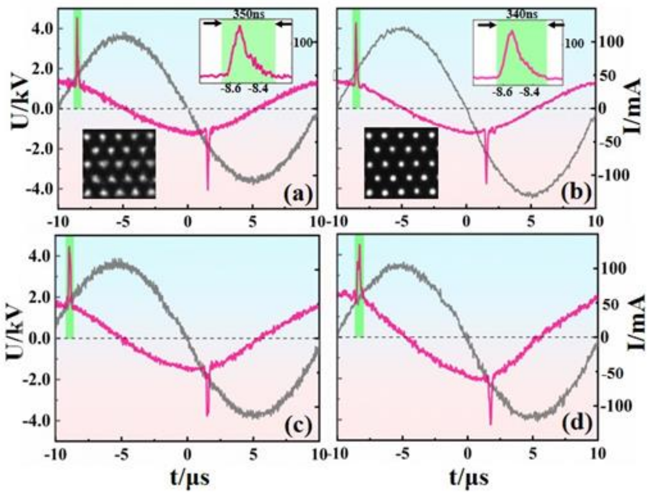

**FIG. S6.** Oscillograms of the discharge current I (pink line) and supply voltage *U* (gray line) during one period of the supply voltage. (a), (c), (d) correspond to the topological triangular PPCs presented in Figs. 6(a)-(c), and (b) corresponds to Fig. 8(a) in the manuscript, respectively. In (a)-(b), the upper insets show closeups of current pulses with the duration marked by a green stripe. The lower insets give the images of instantaneous plasma lattices taken by a high-speed camera. The exposure times (a) $\Delta t$ = 350 ns, (b) $\Delta t$ = 340 ns, and loop 30 times.

We realize both good spatial and temporal control of topological PPCs. In space, the orientation of triangular plasma elements can be manipulated by placing PTFE coatings at different positions on the mesh electrode (Fig. 6 in the manuscript). The sizes of elements can be modulated by changing the discharge parameters including the amplitude of the applied voltage, gas pressure and distance of gas gap. As the supply voltage and gas pressure is increased, the size of elements will be reduced. Moreover, if the distance of gas gap is increased, the size of elements will be increased. In time, all of these discharge elements are ignited simultaneously. As shown in Fig. S6, only one current pulse appears in each half cycle of the supply voltage. This suggests that all of the filaments in this PPC volley within a time window of 350 ns. They repeat the same manner in each half cycle with the jitter of current pulses less than 30 ns. The good temporal periodicity enables one to control the formation of topological PPCs actively by adjusting the voltage frequency.

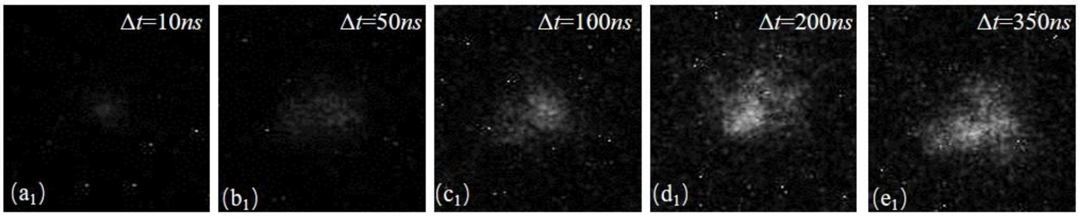


**FIG. S7.** Spatio-temporal resolved measurements for one single plasma element. The images are taken by a high-speed camera with the exposure time $\Delta t = 10ns, 50ns, 100ns\ 200ns\ and\ 350ns$, respectively, in (a$_1$)-(e$_1$).

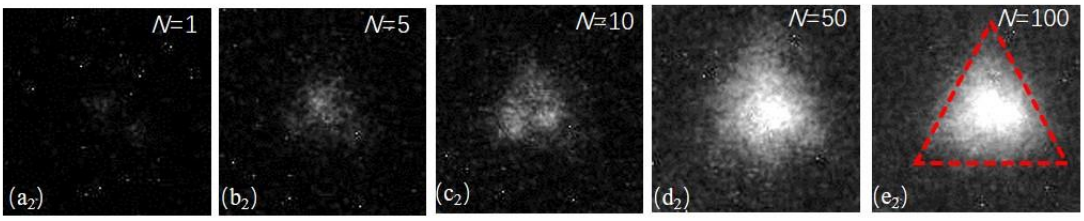


**FIG. S8**. The time-integrated images of the triangular plasma element over different voltage cycles. The images are integrated for 1, 5, 10, 50 and 100 voltage cycles, respectively, in ($a_2$)-($e_2$) with the exposure time $\Delta t$ = 350ns. The red triangles in ($e_2$) indicate the shape of plasma elements**.**

In order to get an understanding of the formation mechanism of triangular plasma elements, the spatio-temporally resolved measurements for one single triangular plasma element have been studied as illustrated in Figs. S7 and S8. It can be seen that only some random discharges emerge when the exposure time $\Delta t \leqslant$ 350ns, which is approximately equal to the duration of the current pulse per half period of A.C. voltage. Besides, the luminance and area of the discharges are increase with the exposure time. The shape of the plasma elements is not triangular in this case, as they appear in the time-averaged images. Fig. S8 shows the images of one single plasma element integrated over 1, 5, 10, 50 and 100 voltage cycles with the exposure $\Delta t$ = 350ns. One can see that the shape of the plasma elements turns triangular with increasing loop times N (Fig. S8 ($e_2$)). Consequently, the formation of triangular plasma elements results from spatio-temporal integration of random filaments over multiple voltage cycles. These filaments distribute in the triangular area located at the three cross points of the hexagon cells of the mesh electrode, where there are no PTFE coatings as shown in Fig. S2.

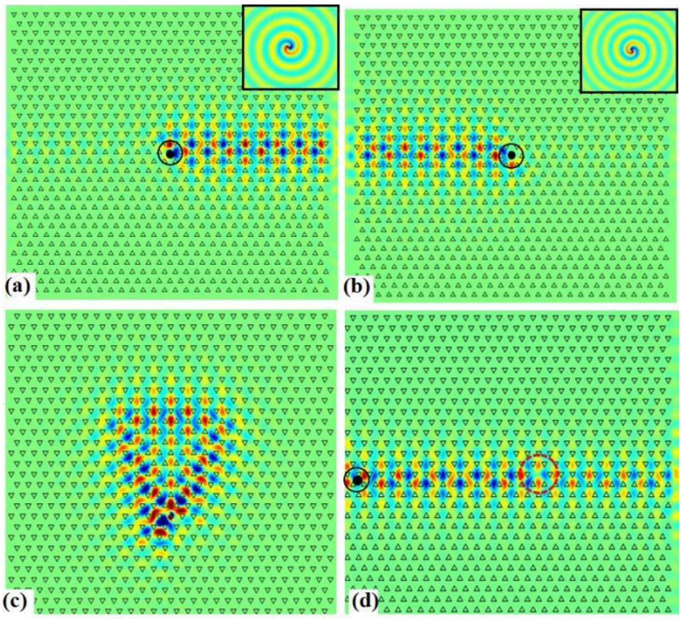


**FIG. S9.** Unidirectional propagation and robustness of the topological edge states. (a)-(d) give $E_Z$ distributions with clockwise decreasing phase of a point-like chiral source, anticlockwise decreasing phase of a point-like chiral source, a triangular micro-cavity along the topological interface, and with presence of a defect along the topological interface, respectively. The frequency of radiated microwave is 35 GHz.

Our 'topological' PPCs have good unidirectional propagation characteristics and robust topological edge states against defects[6], [7]. As shown in Fig. S9(a), point-like chiral sources are constructed by four antennas with phases of 0, π/2, π, 3π/2 to excite valley-dependent topological edge states. A vortex source is placed in the center. When the phases of four antennas decrease clockwise with the optical vortex index $m$ = -1, the electromagnetic energy is transmitted in the right direction along the edge (Fig. S9(a)). Inversely, when the phases of four antennas decrease anticlockwise with the optical vortex index $m$ = 1, the electromagnetic energy is transmitted in the left direction (Fig. S9(b)). Consequently, the unidirectional propagation can be actively controlled by the valley polarization. Owing to this feature, a triangular topology laser based on topological PPCs can be fabricated as shown in Fig. S9(c). Moreover, our PPCs exhibit robust topological edge states[8]. As depicted in Fig. S9(d), the microwaves can still transmit along the interface, although a plasma element has been removed from the

interface. The topological edge states are proven to be immune to the cavities at the interface.

## 5. Measurement of plasma density

The electron density $n_e$ is a key factor to influence the electromagnetic transmission characteristics. In our work, $n_e$ can be estimated by means of the transferred electric charges during the discharge current pulse, which has the form[9]

$$n_e = \frac{\bar{Q}}{enSd} \tag{1}$$

where $e$ is the elementary charge, $S$ is the area of instantaneous filaments, $n$ is the number of plasma filaments, $d$ denotes the distance of discharge gas gap, and $\bar{Q}$ is the averaged transferred electric charges calculated by integration of the current pulses ($\bar{Q} = \int Idt$). For instance, the Line-type topological PPC is ignited during the one current pulse with $\Delta t$ = 350 ns as shown in Fig. S6. Therefore, $\bar{Q}$ can be obtained by integrating the discharge current within this time interval. Meanwhile, the other parameters $n$, $S$ be directly measured by the high-speed camera diagnosis within $\Delta t$. According to Eq. (1), the electron density of plasma lattices can be obtained.

## Reference


[1] Shen, R., L. B. Shao., Wang, B., D. Y. Xing.: Single Dirac cone with a flat band touching on line-centered-square optical lattices. *Physical Review B,* **81**(4), 041410(2010) https://doi.org/10.1103/PhysRevB.81.041410.

[2] Iglovikov, V.I., Hébert, F., Grémaud, B., Batrouni, G.G., Scalettar, R.T.: Superconducting Transitions in Flat Band Systems. *Physical review, B* **90**(9), 094506 (2014) https://doi.org/10.1103/PhysRevB.90.094506.

[3] Pires, A.S.T.: Transport on the ferromagnetic Lieb lattice. *Journal of Magnetism and Magnetic Materials,* **547**(1), 168941 (2022) https://doi.org/10.1016/j.j-mmm.2021.168941.

[4] Zhao, A., Shen, S.-Q.: Quantum anomalous Hall effect in a flat band ferromagnet. *Physical Review B,* **85**(8), 085209(2012) https://doi.org/10.1103/PhysRevB.85.085209.

[5] Kang, Y., Zhong, H., Belić, M.R., Tian, Y., Jin, K., Zhang, Y., Li, F., Zhang, Y.: Conical Diffraction from Approximate Dirac Cone States in a Superhoneycomb Lattice. *Annalen Der Physik* **531**(11), 1900295 (2019) https://doi.org/10.1002/andp.201900295.

[6] Khanikaev, A.B., Mousavi, S.H., Tse, W.-K., Kargarian, M., MacDonald, A.H., Shvets, G.: Photonic topological insulators. *Nat Materials* **12**(3), 233-239(2012) https://doi.org/10.1038/nmat3520.

[7] Li, J., Zhou, C., Yao, J., Yuan, C., Wang, Y., Zhou, Z., Zhang, J., Kudryavtsev, A.A: Valley-dependent topological edge states in plasma photonic crystals. *Plasma Science & Technology* **25**(3), 12-20(2023) https://doi.org/10.1088/2058-6272/ac9347.

[8] Zeng, Y. Chattopadhyay, U. Zhu, B. Qiang, B. Li, J. Jin, Y.: Electrically pumped topological laser with valley edge modes. *Nature* **578**(7794), 246-250 (2020) https://doi.org/10.1038/s41586-020-1981-x.

[9] Fan, W. Hou, X. Jia, M. Tian, M. He, Y. and Liu, F.: Spatiotemporally controllable honeycomb superlattice plasma photonic crystals in dielectric barrier discharge. *New Journal of Physics,* **25**(4), 043003(2023) https://doi.org/10.1088/1367-2630/acc606.
